# Genome-wide identification, classification and expression of lipoxygenase gene family in pepper

**DOI:** 10.1007/s11103-018-0785-y

**Published:** 2018-10-13

**Authors:** Sandeep J. Sarde, Abhishek Kumar, Rahima N. Remme, Marcel Dicke

**Affiliations:** 1 Laboratory of Entomology, Wageningen University, P.O. Box 16, 6700 AA Wageningen, The Netherlands; 2 Division of Molecular Genetic Epidemiology, German Cancer Research Center, Heidelberg, Germany

**Keywords:** Pepper, Lipoxygenases (LOXs), Phylogenetic analysis, Gene transcription, Sequence analyses, Defence

## Abstract

**Key message:**

Lipoxygenases mediate important biological processes. Through comparative genomics, domain-scan analysis, sequence analysis, phylogenetic analysis, homology modelling and transcriptional analysis the lipoxygenase gene family of pepper (*Capsicum annuum*) has been identified.

**Abstract:**

Lipoxygenases (LOXs) are non-heme, iron-containing dioxygenases playing a pivotal role in diverse biological processes in plants, including defence and development. Here, we exploited the recent sequencing of the pepper genome to investigate the *LOX* gene family in pepper. Two LOX classes are recognized, the 9- and 13-LOXs that oxygenate lipids at the 9th and 13th carbon atom, respectively. Using two main in-silico approaches, we identified a total of eight LOXs in pepper. Phylogenetic analysis classified four LOXs (CaLOX1, CaLOX3, CaLOX4 and CaLOX5) as 9-LOXs and four (CaLOX2, CaLOX6, CaLOX7 and CaLOX8) as 13-LOXs. Furthermore, sequence similarity/identity and subcellular localization analysis strengthen the classification predicted by phylogenetic analysis. Pivotal amino acids together with all domains and motifs are highly conserved in all pepper LOXs. Expression of 13-*LOXs* appeared to be more dynamic compared to 9-*LOXs* both in response to exogenous JA application and to thrips feeding. Bioinformatic and expression analyses predict the putative functions of two 13-LOXs, CaLOX6 and CaLOX7, in the biosynthesis of Green Leaf Volatiles, involved in indirect defence. The data are discussed in the context of LOX families in solanaceous plants and plants of other families.

**Electronic supplementary material:**

The online version of this article (10.1007/s11103-018-0785-y) contains supplementary material, which is available to authorized users.

## Introduction

Lipoxygenases (EC 1.13.11.12) are non-heme, iron-containing dioxygenases ubiquitously present in plants, animals and fungi (Brash 1999). In plants, lipoxygenases (LOXs) are well-known to be involved in several plant processes like tuber development, seed germination, fruit ripening and most importantly in plant defences (Bailly et al. 2002; Barry and Giovannoni 2007; Feussner and Wasternack 2002; Kessler 2004; Kolomiets et al. 2001; Yan et al. 2013). Upon insect or pathogen attack, LOXs oxidize polyunsaturated fatty acids (PUFAs) (linoleic acid, α-linolenic acid and arachidonic acid) constituting a (*Z,Z*)-1,4-pentadiene structural unit and catalyzing it into conjugated hydro-peroxides such as oxylipins (Brash 1999; Feussner and Wasternack 2002; Shibata and Axelrod 1995). Oxylipins such as jasmonates, green leaf volatiles (GLVs) and recently discovered death acids, are known for their roles in defence against herbivorous insects and pathogens (Allmann et al. 2010; Bell et al. 1995; Christensen et al. 2015; Losvik et al. 2017; Shen et al. 2014; Yan et al. 2013). Jasmonates and GLVs are 13-LOX-derived products involved in direct and indirect defences, respectively. In indirect defence, GLVs play a pivotal role in the attraction of natural enemies of the herbivores (ul Hassan et al. 2015). Death acids (10-OPDA, 10-oxo-11-phytodienoic acid, and 10-OPEA, 10-oxo-11-phytoenoic acid) are 9-LOX-derived products that in maize (*Zea mays*) accumulate upon southern leaf blight (*Cochliobolus heterostrophus*) infection resulting in the hampering of growth of fungi and herbivorous insects (Christensen et al. 2016, 2015).

Plant LOXs are primarily classified into two major classes, 9- and 13-LOXs, based on their positional specificity to oxygenate linoleic acids (LAs) (Feussner and Wasternack 2002). Moreover, LOXs are also classified as Type-1 and Type-2 based on their primary structure and sequence similarity. LOXs having high sequence similarity (> 75%) among themselves and having no plastidic transit peptide are classified as Type-1, whereas LOXs with moderate sequence similarity (> 35%) and possessing a plastidic transit peptide are classified as Type-2 (Brash 1999; Feussner and Wasternack 2002). All Type-2 LOXs known at present are 13-LOXs, whereas Type-1 LOXs include both 9- and 13-LOXs (Feussner and Wasternack 2002).

Information on LOXs from several plants has been reported. The *Arabidopsis* genome comprises a total of six *LOXs* (*AtLOX1*–*AtLOX6*) (Umate 2011). *AtLOX1* is up-regulated in leaves upon pathogen attack and stress-related hormones (Melan et al. 1993); *AtLOX2* is involved in jasmonic acid (JA) biosynthesis (Bell et al. 1995); *AtLOX3* and *AtLOX4* are essential for flower growth and male fertility (Caldelari et al. 2011); *AtLOX5* is important for lateral root development and defence responses (Vellosillo et al. 2007) and *AtLOX6* is expressed in roots and involved in JA synthesis (Grebner et al. 2013). Among solanaceous plants, different numbers of LOXs are reported in tomato, potato and tobacco. In tomato, *SlLOXA* (*TomLOXA*) and *SlLOXB* (*TomLOXB*) are induced during fruit ripening (Ferrie et al. 1994; Griffiths et al. 1999); *SlLOXC* (*TomLOXC*) participates in production of flavour compounds resulting from fatty acids (Chen et al. 2004); *SlLOXD* (*TomLOXD*) is involved in wound-induced JA biosynthesis, enhancing resistance against herbivores and pathogens (Yan et al. 2013); *SlLOXE* (*TomLOXE*) is expressed in breaker fruit (Chen et al. 2004) and *SlLOXF* (*TomLOXF*) enhances systemic resistance stimulated by *Pseudomonas putida* BTP1(Mariutto et al. 2011). In tobacco, *NaLOX1* codes for a 9-LOX and is specifically expressed in roots (Allmann et al. 2010); *NaLOX2* is involved in biosynthesis of GLVs (Allmann et al. 2010; VanDoorn et al. 2010); and *NaLOX3* is involved in JA biosynthesis (Halitschke and Baldwin 2003; Kessler 2004). Furthermore, in potato, *StLOXH1* mediates the biosynthesis of volatile C6-aldehydes (GLVs) involved in defence (Leon et al. 2002) and *StLOXH3* is involved in the JA biosynthetic pathway (Royo et al. 1996). Knowledge on LOXs has also been presented in grapevine (Podolyan et al. 2010), kiwifruit (Zhang et al. 2006), rice (Umate 2011), apple (Vogt et al. 2013), soybean (Shin et al. 2008), cucumber (Liu et al. 2011), and olive (Padilla et al. 2009, 2012).

Pepper (*Capsicum annuum*) is an economically important crop worldwide. It is used e.g. as food, spice and in pharmacology. There are many biotic and abiotic factors constraining pepper production (Kulkarni and Phalke 2009; Kurunc et al. 2011; Pakdeevaraporn et al. 2005; Shipp et al. 1998). Despite increasing commercial significance of pepper, the molecular mechanisms underlying different plant processes are still unknown. For instance, to develop resistance against pathogens and insects, identifying genes involved in different defence mechanisms in pepper is important.

To date, no comprehensive knowledge on the pepper *LOX* gene family is available. One 9-LOX, *CaLOX1*, involved in defence and cell-death responses against pathogens has been reported (Hwang and Hwang 2010). Recently, a second member of the *LOX* gene family (*CaLOX2; Capana03g000103*) was identified, playing a role in JA-regulated defence against Western flower thrips (*Frankliniella occidentalis*) (Sarde et al. 2018). Therefore, there is a need of a genome-wide survey of the *LOX* gene family of pepper. Here, we performed comparative genomics and domain-scan analyses for identification and classification of the *LOX* gene family in pepper. To investigate the conservation levels of pepper LOXs compared to known LOXs of other plant species, we subjected pepper LOXs to sequence analysis, phylogenetic analysis and homology modelling. Furthermore, to investigate the role of pepper *LOXs* in defence mechanisms, we examined their expression upon two treatments: exogenous JA application and exposure to feeding by a natural inducer of JA, the cell-content feeding insect Western flower thrips (WFT). WFT was selected because it is a major pest on pepper and well-known to induce JA signaling (Hickman et al. 2017; Steenbergen et al. 2018). The resulting data provide insights into putative functions of these genes in pepper.

## Materials and methods

### Sequence acquisition and identification of pepper LOXs

Protein sequences of tomato (*Solanum lycopersicum*) lipoxygenases were obtained from the Ensembl Plants database (http://www.ensembl.org) (Yates et al. 2016). LOX sequences from *Brassica oleracea, Brassica napus, Brassica rapa, Arabidopsis thaliana, Nicotiana attenuata, Nicotiana tabacum, Solanum tuberosum, Zea mays* and *Actindia deliciosa*, were downloaded from NCBI (http://www.ncbi.nlm.nih.gov/). *Oryza sativa* and *Cucumis melo* LOX sequences were retrieved from the Rice Genome Annotation Project (http://rice.plantbiology.msu.edu/) and the Melonomics database (http://melonomics.net/), respectively. Two main approaches were used for the identification of the pepper *LOX* gene family. First, BLAST searches were performed locally on the *Capsicum annuum* L. *Zunla-1* proteome (Qin et al. 2014) using Tomato LOX proteins as queries. Second, the *Capsicum annuum* L. *Zunla-1* proteome was entirely analyzed for the presence of lipoxygenase gene family signature domains, LOX and PLAT/LH2 (polycystin-1, lipoxygenase, α-toxin domain or lipoxygenase homology) using the Pfam database (v27.0) in the CLC Bioworkbench (https://www.qiagenbioinformatics.com/).

### Sequence alignment of lipoxygenases

Alignment of LOX protein sequences was performed using the MUSCLE tool (Edgar 2004) with default settings. Editing and visualization of alignment was produced in GENEDOC (Nicholas et al. 1997). Sequence logos of conserved regions in pepper LOX proteins were generated by Weblogo 3.3 (Crooks et al. 2004).

### Phylogenetic analysis of plant LOXs

Seventy-two plant LOX protein sequences were analyzed, including one known pepper LOX, CaLOX1(L) (*L* stands for ‘literature’) (Hwang and Hwang 2010) and eight pepper LOXs identified in the present study. A Maximum likelihood tree using WAG-model (Hall 2013), with 1000 bootstrap replicates was generated using MEGA 7.0 (Kumar et al. 2016). The tree was edited with the Figtree tool (http://tree.bio.ed.ac.uk/software/figtree/).

### Sequence analysis and identification of conserved sequences

Conserved sequences and pivotal amino acids were identified by manual observations on pepper LOX alignments in GENEDOC (Nicholas et al. 1997). Molecular weight and isoelectric point of pepper LOX proteins were calculated by protein isoelectric point calculator (Kozlowski 2016). Subcellular localization analysis was performed using TargetP 1.1 (http://www.cbs.dtu.dk/services/TargetP/).

### Homology modeling of CaLOX1 and CaLOX2 protein

We generated a protein structural model of CaLOX1 and CaLOX2 using the I-TASSER (Roy et al. 2010) database and the resulting model was visualized with YASARA (Krieger et al. 2002).

### Plant growth conditions, thrips rearing and bioassays

Sweet pepper [*Capsicum annuum* (Mandy variety, Rijk Zwaan (De Lier, The Netherlands))] plants were grown in a greenhouse at 23–25 °C, 70 ± 10% relative humidity and 16L:8D photoperiod. Four-week-old plants were used in the experiments for both treatments. Western flower thrips (WFT; *Frankliniella occidentalis*) were reared on bean pods (*Phaseolus vulgaris*) in a climate-controlled cabinet (25 ± 2 °C, 70 ± 10% relative humidity, L16:8D photoperiod). For thrips treatment in the gene expression experiment, five 2nd instar thrips larvae were placed in clip cages and used for infestation on one of the first two true leaves. Empty clip cages were used on control plants for each time point. Samples were harvested at 0, 2, 4, 6, 8, 10 and 24 h post infestation, frozen in liquid nitrogen and stored at − 80 °C.

### RNA extraction and qRT-PCR

Transcriptional responses of pepper *LOXs* in response to JA treatment (100 µM) and thrips feeding were assessed by qRT-PCR. For JA-treatment, plants were dipped in 100 µM of JA (treatment) or mock-treated with water (control), both mixed with 0.1% of Tween20. One of the first two true leaf samples were harvested at 0, 0.5, 1, 2, 3, 6, 8, 10 and 24 h post JA application, frozen in liquid nitrogen and stored at − 80 °C. For both treatments (JA and thrips), control samples were harvested at each time point to rule out the effect of circadian rhythm on the expression of *LOX* genes. Four to five biological replicates (individual plants) were harvested and analysed for each time point and treatment. Each biological replicate comprises one individual plant. Bioline kit (ISOLATE II RNA Plant Kit), in accordance to its protocol, was used for RNA extraction. cDNA synthesis was executed with 1 µg of total RNA with Bio-Rad iScript cDNA synthesis kit. For qPCR, a reaction mixture comprising of 12.5 µl of SYBR Green (Bioline), 1 µl (10 µM) of forward and reverse primers, 5.5 µl RNase free-water and 5 µl cDNA was used. The data normalization was performed with a reference gene, *CaActin*. The PCR cycle conditions used were 95 °C for 3 min, followed by 40 cycles of 95 °C for 15 s, and 60 °C for 45 s. Melt curves for each gene were recorded at the end of each cycle. All primers used for qPCR are presented in Supplementary file S1.

Relative gene expression was studied using the geometric mean of Ct (threshold cycles) values (Vandesompele et al. 2002) from the reference gene *CaActin* using the 2–^ΔΔCt^ method (Livak and Schmittgen 2001).

### Statistical analysis

The gene expression data were subjected to a Student’s *t*-test.

## Results and discussion

### Identification of lipoxygenase gene family in pepper

A genome-wide search for lipoxygenase genes in pepper was performed by implementing two main approaches: homology search and scanning of the pepper proteome for the presence of “lipoxygenase” and “PLAT/LH2” domains. Both approaches resulted in the identification of eight lipoxygenases in the *Capsicum annuum* L. *Zunla-1* proteome (Table 1). Several proteins depicting the presence of either one lipoxygenase domain or the PLAT/LH2 domain were excluded from analysis based on arguments of Chen et al. (2015).

**Table 1:** Characteristics of lipoxygenase genes in pepper

Name	Gene IDs	Location coordinates	Nucleotide			Protein			Predicted LOX class	References
			Chr.	No. of Introns	ORF length (bp)	Length (a.a)	Mol. wt. (Da)	PI		
*CaLOX1*	Capana01g003855	261291828:261298825	1	8	2502	833	94,198	5.40	9	–
*CaLOX2*	Capana03g000103	1425452:1429795	3	7	2733	910	102,696	6.33	13	(Sarde et al. 2018)
*CaLOX3*	Capana03g003732	235908782:235912945	3	8	2574	857	97,476	5.83	9	–
*CaLOX4*	Capana03g003733	235924316:235929152	3	8	2637	878	99,969	5.56	9	–
*CaLOX5*	Capana01g000180	2592639:2595818	1	8	2463	820	92,135	5.78	9	–
*CaLOX6*	Capana01g001574	52477015:52494659	1	8	2379	792	89,959	5.70	13	–
*CaLOX7*	Capana01g001578	52720202:52733587	1	9	2664	887	99,998	5.62	13	–
*CaLOX8*	Capana11g000928	64997805:65002262	11	8	2748	915	104,131	7.54	13	–

The total number of LOXs in pepper (8) is similar to that in tomato (7). This number is also close to the number in *Arabidopsis* (6) (Umate 2011) and kiwifruit (6) (Zhang et al. 2006), double the number in olive (4) (Padilla et al. 2009, 2012) and much lower than in melon (18) (Zhang et al. 2014), cucumber (23) (Liu et al. 2011) and grapevine (18) (Podolyan et al. 2010). This diverse number of LOXs in different plant species indicates that this gene family has not been conserved during evolution, despite similarities in biochemical functions of the gene family in different plant species (Feussner and Wasternack 2002).

Genomic and proteomic features of the pepper *LOX* gene family do not differ much (Table 1). At the genomic level, the number of introns varies between 7 and 9, whereas, ORF (Open Reading Frame) length ranges from a minimum of 2379 bp to a maximum of 2748 bp. Most of the pepper *LOXs* are located on Chromosomes 1 and 3, with the exception of *CaLOX8* (Capana11g000928) on Chromosome 11. At the protein level, LOX length varied between 792 and 915 aa, the predicted isoelectric point (PI) ranged between 5.4 and 7.5 and the predicted molecular weight of the proteins ranged from 89,959 to 104,131 Da. Sequence comparison among pepper LOXs at the protein level shows high sequence identity (33–70%) and similarity (48–77%) (Table 2). Taken together, these genomic and proteomic features show a close relation among the pepper *LOXs*, indicative of a gene family.

**Table 2:**
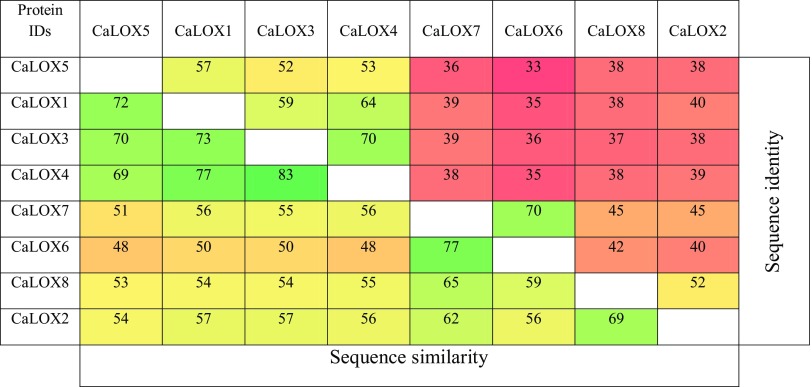
Pepper LOX protein identities and similarities (%)

High, intermediate and low similarity/identity of genes is shown in green, yellow and red color, respectively

### Phylogenetic analysis of lipoxygenases

**Fig. 1:**
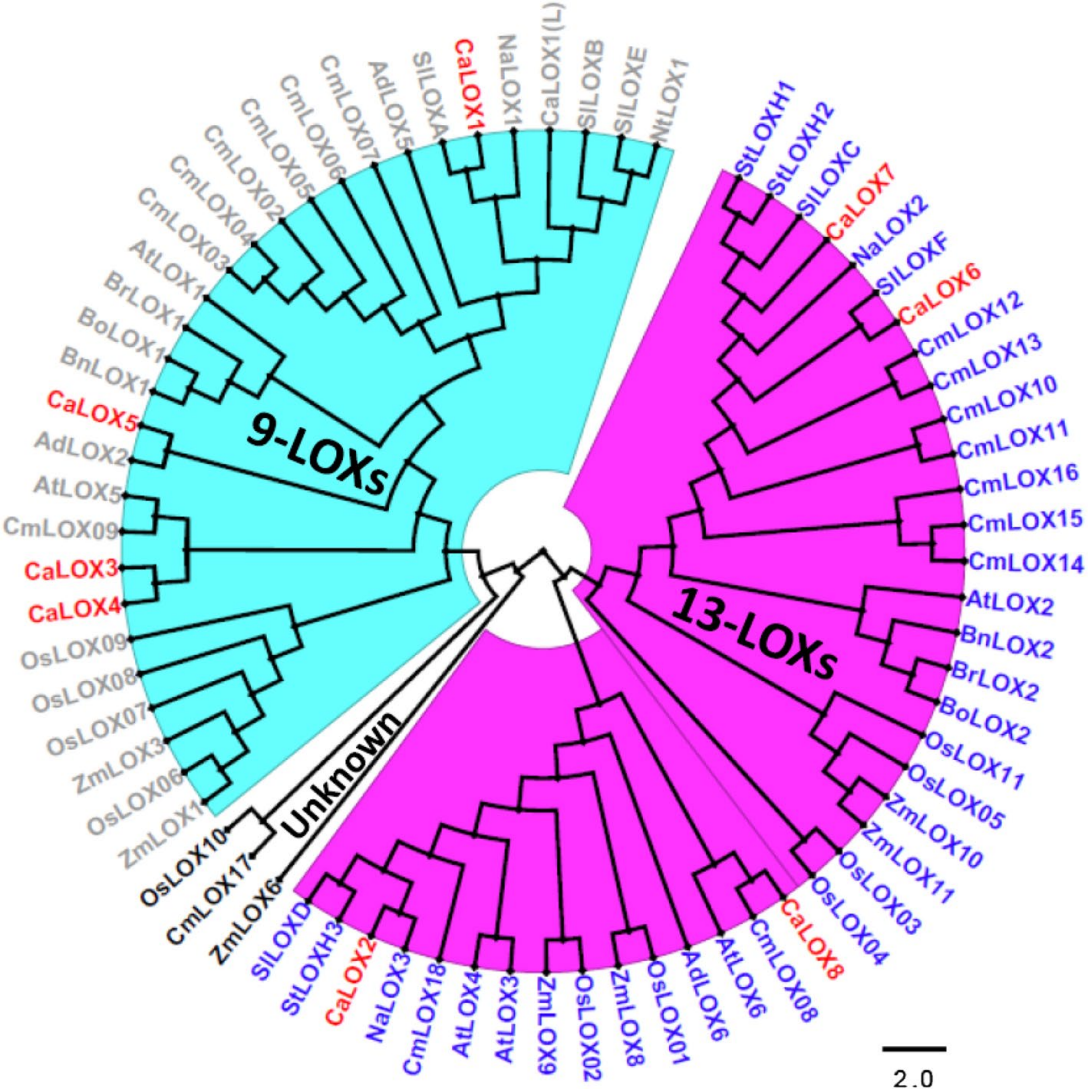
Phylogenetic analysis of plant lipoxygenases. The evolutionary relationship between pepper and other LOX proteins. The tree was generated by MEGA 7 using Maximum Likelihood method with 1000 bootstraps and viewed in Figtree. The scale bar represents the branch length. Different classes of LOXs are depicted in different colors, 13-LOXs in purple; 9-LOXs in blue; unclassified without color. Identified pepper LOXs are highlighted in red color. Species abbreviations used for phylogeny are as follows. At: *Arabidopsis thaliana*, Bo: *Brassica oleracae*, Bn: *Brassica napus*, Br: *Brassica rapa*, Sl: *Solanum lycopersicum*, St: *Solanum tuberosum*, Na: *Nicotiana attenuata*, Nt: *Nicotiana tabacum*, Ca: *Capsicum annuum*, Os: *Oryza sativa*, Zm: *Zea Mays*, Cm: *Cucumis melo*, Ad: *Actindia deliciosa*

To determine the evolutionary relationship and predict the classification of pepper LOXs, a maximum-likelihood phylogenetic tree with 1000 bootstraps was generated. For this, we used sixty-four previously known plus eight pepper LOX protein sequences from twelve different plant species, comprising monocots and dicots. The tree explicitly categorizes plant LOXs into 9-LOXs, 13-LOXs and uncharacterized LOXs. From the identified eight pepper LOXs, four LOXs (CaLOX1, CaLOX3, CaLOX4 and CaLOX5) are characterized, including the previously described CaLOX1(L) (Hwang and Hwang 2010) into the 9-LOX group and four other LOXs (CaLOX2, CaLOX6, CaLOX7 and CaLOX8) into the 13-LOX group (Fig. 1). Moreover, upon closer examination of the 9- and 13-LOXs major clades, explicit sub-clades of monocot and dicot LOXs are formed indicating that this gene family has evolved differently in monocots and dicots (Fig. 1).

In the 13-LOX clade, pepper LOXs group with well-characterized Solanaceae 13-LOXs like SlLOXD, StLOXH3, NaLOX3, SlLOXF, NaLOX2, StLOXH1 and SlLOXC (Fig. 1). These clusters or sub-clusters among known LOXs and newly identified LOXs may be useful to predict biochemical features and molecular functions of the newly identified pepper LOXs. CaLOX2 clusters with SlLOXD, StLOXH3 and NaLOX3, well-known to be involved in JA biosynthesis (Halitschke and Baldwin 2003; Kessler 2004; Royo et al. 1996; Yan et al. 2013) suggesting that CaLOX2 has a similar function. This matches with our recent study experimentally confirming that *CaLOX2* is involved in JA biosynthesis upon thrips feeding (Sarde et al. 2018). Virus-induced gene silencing of *CaLOX2* led to disruption of the jasmonate pathway resulting in enhanced performance of thrips. CaLOX6 clusters with SlLOXF, known to be involved in systemic resistance to Pseudomonas putida BTP1 (Mariutto et al. 2011). CaLOX7 groups with NaLOX2, StLOXH1 and SlLOXC. These three LOXs are involved in the biosynthesis of green leaf volatiles (Allmann et al. 2010; Chen et al. 2004; Leon et al. 2002; VanDoorn et al. 2010). CaLOX8 seems to be related to AtLOX6, known to provide resistance against biotic and abiotic stresses through oxylipin biosynthesis in roots (Grebner et al. 2013).

Also in the 9-LOX clade, pepper LOXs cluster with functionally characterized LOXs of other plant species such as AtLOX5, AdLOX2, SlLOXA, SlLOXB (Fig. 1). AdLOX2, that mediates the generation of C6 aldehydes in kiwifruit (Zhang et al. 2009), clusters with CaLOX5, suggesting that CaLOX5 has a similar function. CaLOX3 and CaLOX4 form a major clade with AtLOX5 and CmLOX09. AtLOX5 is involved in lateral root development and defence responses (Vellosillo et al. 2007). Additionally, relatedness of CaLOX3 and CaLOX4 to each other, suggests that they may be isoforms mediating the same biological process. Furthermore, clustering together of identified CaLOX1 from Capsicum annuum Zunla-1 proteome (Qin et al. 2014) and known CaLOX1(L) reflects their similarity/relatedness, suggesting them to be the same protein. Hwang and Hwang (2010) identified *CaLOX1(L)* independently from cDNA clones and reported it to be involved in defence and cell-death responses against pathogens. Furthermore, CaLOX1 identified here and the previously reported CaLOX1(L) (Hwang and Hwang 2010) cluster with LOXs like SlLOXA and SlLOXB, two LOXs that are up-regulated in ripening tomato fruits (Ferrie et al. 1994; Griffiths et al. 1999). Nevertheless, taken together, the predicted functions of pepper LOXs require further experimental validation to characterize their molecular functions, as reported for CaLOX1(L) and CaLOX2 (Hwang and Hwang 2010; Sarde et al. 2018).

Finally, the reported uncharacterized LOXs like OsLOX10, CmLOX17 and ZmLOX6 clearly form an outgroup from the 9- and 13-LOXs (Cao et al. 2016; Liu et al. 2011; Zhang et al. 2014).

### Sequence analysis consolidates phylogenetic classification of pepper LOXs

The lipoxygenase family of pepper (CaLOX1–CaLOX8) is highly conserved with variable sequence identities and similarities with each other (Table 2). It is known that, based on degree of sequence similarity and presence/absence of chloroplast-transit peptide, LOXs are classified into Type-1 or Type-2 (Feussner and Wasternack 2002; Porta and Rocha-Sosa 2002). Type-1 LOXs show high sequence similarity (> 75%) in the absence of a chloroplast-transit peptide; in contrast, Type-2 LOXs show low sequence similarity and the presence of a chloroplast-transit peptide. The pepper LOXs CaLOX1 and CaLOX3, CaLOX4 and CaLOX5, exhibit high sequence similarity (> 70%) and identity (> 52%) with each other compared to other LOXs. In contrast, CaLOX2 and CaLOX6, CaLOX7 and CaLOX8 show low sequence similarity among themselves with the exception of CaLOX6 and CaLOX7. CaLOX6 and CaLOX7 show high sequence similarity and identity among themselves, but not when compared to the rest of the LOXs, suggesting that CaLOX6 and CaLOX7 are isoforms of each other. Furthermore, the presence of a chloroplast-transit peptide in sequences of CaLOX2 and CaLOX6, CaLOX7 and CaLOX8 suggests that they are localized in the chloroplast. Collectively, sequence similarity and sub-cellular localization analysis indicates classification of CaLOX1, CaLOX3, CaLOX4 and CaLOX5 into Type-1 and CaLOX2, CaLOX6, CaLOX7 and CaLOX8 into Type-2.

Furthermore, plant LOXs are also classified into 9- and 13-LOXs, based on their positional specificity of action on the substrate (Feussner and Wasternack 2002). The presence of Phe608/His608 or Val608 residue predicts LOX activity as 13- or 9-LOX, respectively. Multiple sequence alignment of all pepper LOXs clearly shows the occurrence of valine in CaLOX1 and CaLOX3, CaLOX4 and CaLOX5 classifying them as 9-LOXs and phenylalanine in CaLOX2 and CaLOX6, CaLOX7 and CaLOX8 classifying them as 13-LOXs (Fig. S1). This agrees with the observation that all Type-2 LOXs identified so far are 13-LOXs (Feussner and Wasternack 2002).

Therefore, both classification methods provide consensus on distribution of pepper LOXs into different classes, thus consolidating our methodology and predictions. Moreover, it also suggests to use the parameters from both approaches in the future for classification of plant lipoxygenases.

### High conservation of motifs and pivotal amino acids

**Fig. 2:**
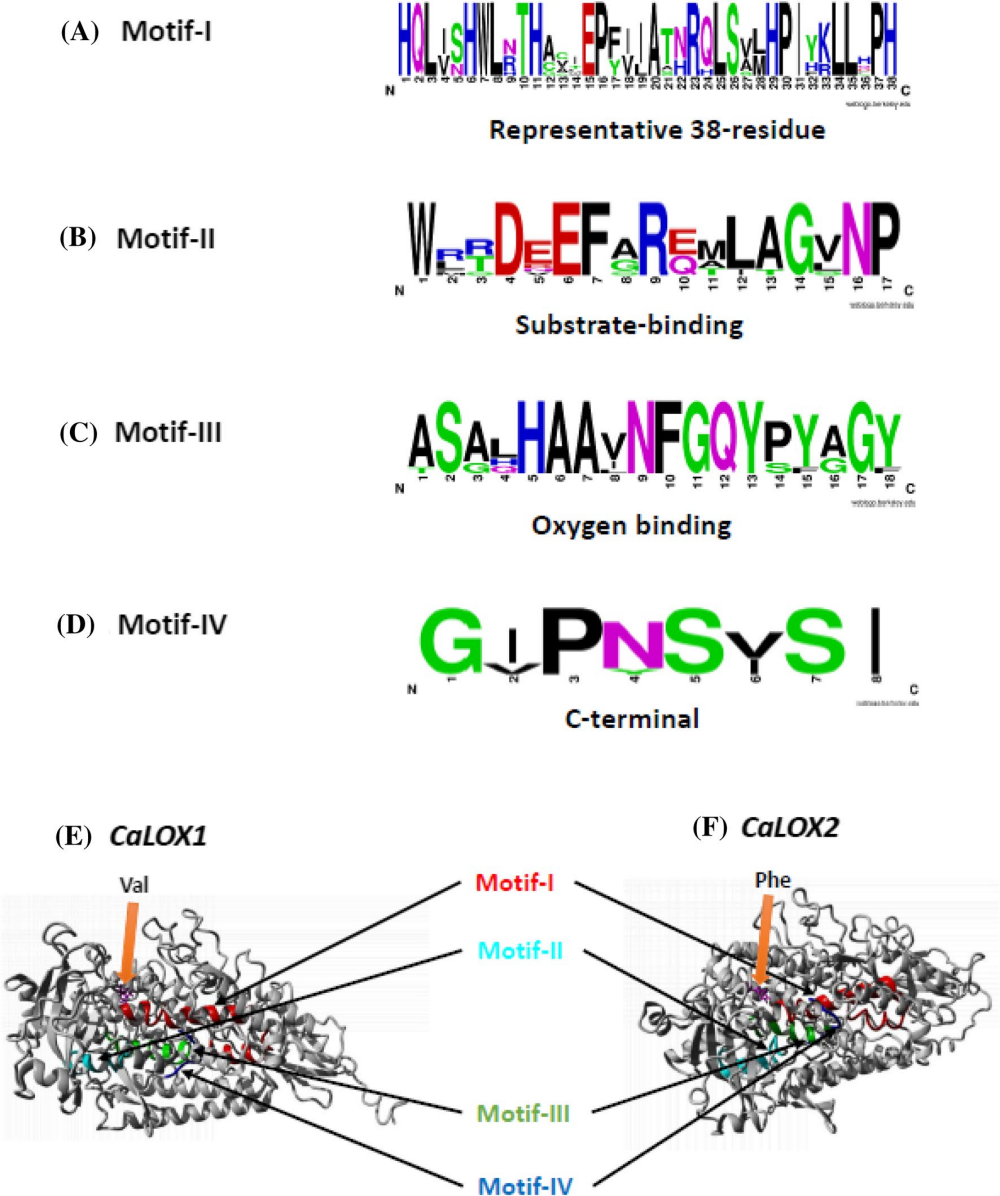
Conservation of sequence motifs in pepper lipoxygenases (**a**–**d**) and protein models of *CaLOX1* and *CaLOX2* (**e**–**f**). Highly representative 38-residue motif (**a**), substrate binding (**b**), oxygen binding (**c**) and C-terminal (**d**) motifs are highly conserved. Protein model of a 9-LOX *CaLOX1* (**e**) and 13-LOX *CaLOX2* (**f**) depicting conservation of highly representative 38-residue (red), substrate binding (cyan), oxygen binding (green) and C-terminal motif (blue) motifs. 13- or 9-LOX activity determinant Phe or Val residue, respectively are depicted

Lipoxygenases are characterized by the presence of a 38-residue representative sequence, a substrate-binding domain, an oxygen binding domain and a C-terminal motif (Padilla et al. 2009, 2012). The highly representative 38-residue sequence in lipoxygenases is important for stability of lipoxygenases. In addition, the enzymatic activity or efficiency of lipoxygenases can be severely affected if any of the residues of this sequence is substituted (Chen et al. 2015). This sequence is highly conserved in all the predicted pepper LOXs (Fig. 2a, e, f). Also, the other motifs like substrate binding, oxygen binding and the C-terminal are conserved among all pepper LOXs (Fig. 2a–f).

Among the conserved amino acids, the three histidine residues (including two from the representative 38-residue sequence) His499, His504, His690 with Asn694 and Ile839 are identified to be vital for binding to non-heme iron (Boyington et al. 1997; Feussner and Wasternack 2002; Padilla et al. 2012; Porta and Rocha-Sosa 2002; Steczko et al. 1992). All these five amino acids appear to be conserved in the pepper LOXs (Fig. S1) with an exception for Ile839 in CaLOX7. Substitution of C-terminal isoleucine with any other amino acid except valine led to inactivation of lipoxygenases, whereas, substitution with valine had positive consequences for enzymatic activity (Chen et al. 1994). Therefore, the absence of a C-terminal motif and the presence of Ile839, essential for non-heme iron binding, leads us to suggest that CaLOX7 may have an altered enzymatic activity. Moreover, the conserved Val608 or Phe608/His608 residue that are indicative for 9- or 13-LOX activity, respectively (Hornung et al. 1999; Padilla et al. 2009, 2012; Sloane et al. 1991), are found highly conserved in pepper LOXs (Fig. 2e, f and Fig. S1). The determinant residues for inverse substrate orientation and S-stereospecificity of LOXs, Arg and Ala, respectively (Coffa and Brash 2004; Hornung et al. 1999) are well-conserved as well in pepper LOXs (Supplemental Fig. S1). Taken together, the conservation of motifs and pivotal amino acids suggests that functions of pepper LOXs are conserved to their respective homologs in other plant species.

### Expression pattern of lipoxygenases upon JA application and thrips feeding

**Fig. 3:**
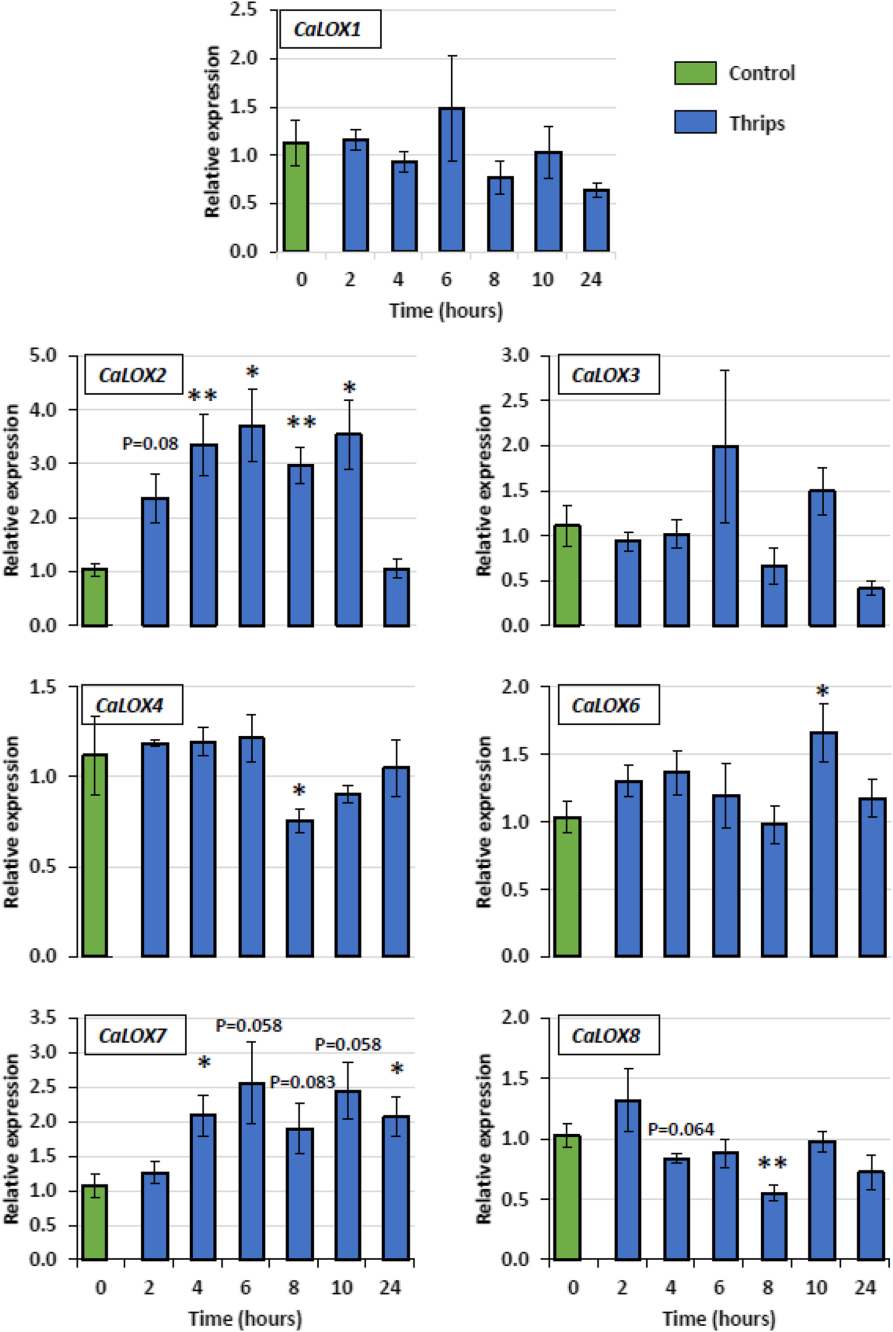
Quantitative RT-PCR (RT-qPCR) of pepper lipoxygenase genes in sweet pepper leaves in response to thrips (*F. occidentalis*) feeding. Five 2nd instar thrips larvae in a clip cage fed on the first true leaf of 4-week-old pepper plants. Clip cages without thrips were used on control plants. Expression of the housekeeping gene *CaActin* was used to normalize the expression level of each *LOX* gene at each time point. Relative expression compared to the control for the same time point is presented. Bars represent means ± SE of 4–5 biological replicates. Bars marked with asterisks indicate significant differences (Student’s *t*-test) to corresponding control samples for the same time point, *P < 0.05, **P < 0.01. For bars without asterisk or P value, the P value is > 0.10

qRT-PCR was performed to investigate the expression dynamics of pepper *LOXs* over time upon thrips feeding or exogenous JA application. Upon thrips feeding, two 13-*LOXs* (*CaLOX2* and *CaLOX7*) are up-regulated for most of the analyzed time points (Fig. 3). Induction of *CaLOX2* occurred after 2 h of thrips feeding and remained up-regulated. This gene’s involvement in JA biosynthesis has been experimentally supported (Sarde et al. 2018). *CaLOX7* is significantly up-regulated at 4 h after the start of feeding and remained up-regulated throughout the period suggesting that it may have a role in defence. *CaLOX6* is significantly up-regulated after 10 h of feeding. In contrast, all other *LOXs*, i.e. *CaLOX1, CaLOX3, CaLOX4* and *CaLOX8* did not show induction over time (Fig. 3). *CaLOX4* and *CaLOX8* are significantly down-regulated after 8 h of thrips feeding. *CaLOX5* expression is not shown due to its unstable expression resulting in a high degree of variation. This instability of *CaLOX5* expression was also confirmed by its expression pattern in an RNA-seq dataset (Sarde et al. unpublished data).

**Fig. 4:**
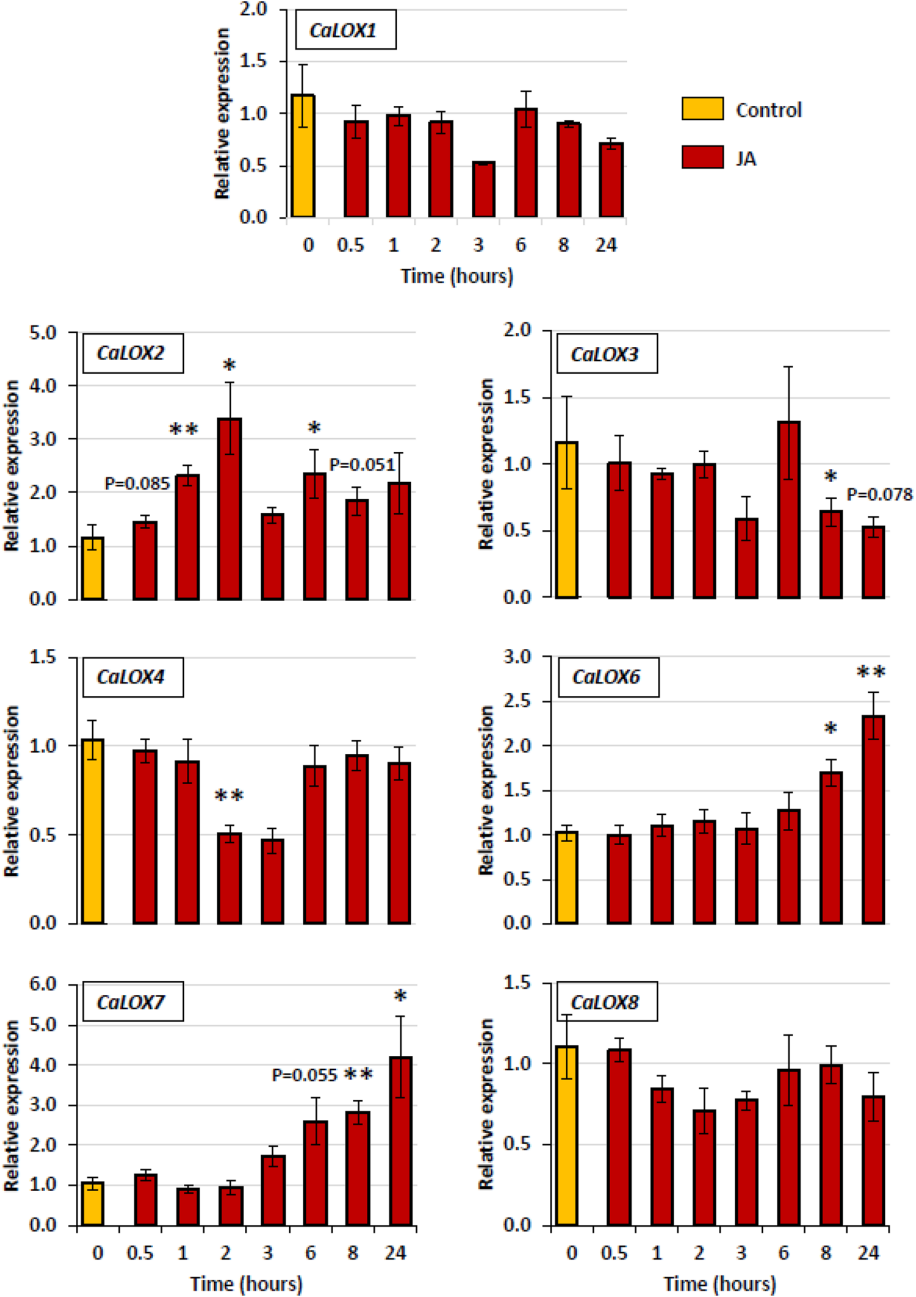
Quantitative RT-PCR (RT-qPCR) of pepper lipoxygenase genes in sweet pepper leaves in response to exogenous JA application. Pepper plants were dipped in water + Tween20 (control) or 100 µM JA + Tween20 (treatment). Expression of the housekeeping gene *CaActin* was used to normalize the expression level of each *LOX* gene at each time point. Relative expression compared to the control for the same time point is presented. Bars represent means ± SE of 4–5 biological replicates. Bars marked with asterisks indicate significant differences (Student’s *t*-test) to corresponding control samples for the same time point, *P < 0.05, **P < 0.01. For bars without asterisk or P value, the P value is > 0.10

In *Arabidopsis*, it is well-known that *LOX* expression is triggered following application of JA due to presence of a positive feedback loop that amplifies JA responses (Hickman et al. 2017). Upon exogenous JA application, *CaLOX2*, known to be involved in JA biosynthesis (Sarde et al. 2018), shows significant induction after 2 h which was maintained until 6 h after JA application with exception at 3 h (Fig. 4). This instant up- and downregulation of *CaLOX2*, suggests involvement of some feedback mechanism in JA-biosynthetic pathway. *CaLOX6* and *CaLOX7* are also upregulated upon JA application. Both of them exhibit high expression levels at similar timepoints i.e., 8 h and 24 h after JA application. In contrast, the other *LOX* genes, i.e. *CaLOX1, CaLOX3, CaLOX4* and *CaLOX8*, were not up-regulated at any time point, but exhibited down-regulation at several time points (Fig. 4).

In general, the 9-*LOXs* in pepper (*CaLOX1, CaLOX3* and *CaLOX4*) did not show any induction, but rather down-regulation at certain time points in both treatments, i.e. JA application and thrips feeding. This fits to the fact that 9-*LOXs* are especially involved in functions such as plant–pathogen interactions, storage of proteins and tuber development (Feussner and Wasternack 2002). In contrast, the 13-*LOXs* were more responsive to both treatments, except *CaLOX8*. Similarity of CaLOX7 to NaLOX2 and SILOXC (Fig. 1), both known to be involved in the biosynthesis of green leaf volatiles (GLVs) (Allmann et al. 2010; Chen et al. 2004; Shen et al. 2014; VanDoorn et al. 2010), and it’s up-regulation upon both thrips feeding and JA application (Figs. 3, 4) suggest a role of CaLOX7 in the biosynthesis of GLVs in pepper. Additionally, in tomato *SILOXC*-antisense lines, low expression of both *SILOXC* and *SILOXF* resulted in decreased levels of C5 and C6 leaf volatiles, suggesting a possible synergistic involvement of *SILOXC* and *SILOXF* in the biosynthesis of these plant volatiles (Shen et al. 2014). Therefore, the similarity of CaLOX6 to SILOXF (Fig. 1) and its induction upon both JA application and thrips feeding makes it a potential candidate to test for its synergistic role with CaLOX7 in volatile biosynthesis (Figs. 3, 4).

In conclusion, this study has identified and classified eight LOXs in pepper. Phylogenetic analysis classified four LOXs as 9-LOXs (CaLOX1, CaLOX3, CaLOX4 and CaLOX5) and four others as 13-LOXs (CaLOX2, CaLOX6, CaLOX7 and CaLOX8) with predictions of their putative functions. Pepper LOX proteins are highly conserved in all lipoxygenase characteristics. Characterization of *CaLOX2* encoding for a LOX that is involved in JA biosynthesis is confirmed by a recent experimental study through a combination of in-silico, transcriptional, behavioural, and chemical analyses plus silencing of *CaLOX2* through virus-induced gene silencing (Sarde et al. 2018). For the other LOXs their function remains to be elucidated. High expression levels of 13-*LOXs* in pepper with support of in-silico analysis predict potential candidate genes (*CaLOX6* and *CaLOX7*) that code for enzymes involved in GLV biosynthesis in pepper. Finally, this comprehensive study provides a pepper *LOX* genes repository to further elucidate their functional roles in respective biological processes.

## Electronic supplementary material

Below is the link to the electronic supplementary material.

Supplementary material 1 (PDF 272 KB)

Supplementary material 2 (XLSX 9 KB)
