# Supplementary material for: Genome-wide identification, classification and expression of lipoxygenase gene family in pepper
Source: Plant Mol Biol. 2018 Oct 13;98(4):375–87. doi: 10.1007/s11103-018-0785-y (PMC6244800; doi:10.1007/s11103-018-0785-y)
Supplement: Supplementary file 1 — Supplementary material 1 (PDF 272 KB) [file 11103_2018_785_MOESM1_ESM.pdf]

```
CaLOX1    : -------MIKNLVDGLIHHDSSKK------------------------------------------------------------------ :  17
CaLOX2    : MALAKEIMGISLLEK-----SSSS------SMALLNSNLNQKENQLWFNHQFPGRRNLRTRKAFRQCTMAA---ISENLIKVVPEKAVKFK :  77
CaLOX3    : ------MGCTEILEKLLETVCGKQ-----------------------HDVRIERDPSNGTSSIK------------------------- :  35
CaLOX4    : -MGVFPLCSTEMLEKLLNVVCGRN-----------------------HDTTDENNCNNNHTNGKK------------------------ :  41
CaLOX5    : -----------------------------------------------------------MASKGKKQM--------------------- :   9
CaLOX6    : -MLKPHLQQSSQSSNTLIPWNTKP----SFPINILNKNFRIKNK-----KSFRVH--YNNVANSTKAVLSTTTTEKSTLVQSTNENSTS-- :  77
CaLOX7    : -MLKLHLQQSPQSTKPLIPWSTKPISLASFPINVLNENFGIKNKN----NNFRIHHNYNNAANSTKAVLST------------EKSTG-- :  71
CaLOX8    : MMFTAQPRSTPLSSEIQHVFTVRS------PVEIMTTRRKISSQI----SRFKVKAVIQSGNEEKKTVESGKLVEKSGEESNGFSGKGVR :  81
CaLOX1(L) : ----------MLLEKIVDVISGKN-------------------------------------DDGKK----------------------- :  19

CaLOX1    : -VKGTVVMMKKNALDFTDLAGSLTDKLFEALGQKVSLQLI-SSVQGDP--------------------------GESAFGVTFD-WN-EEF :  78
CaLOX2    : -VRAVVTVRNKNKEDLKETIVKHLDAFTDKIGRNVALELI--SIDIDPNTK-GPKRSNQAVLKDW-SKKSNLKTERVNYTAEFI-VD-SNF : 161
CaLOX3    : -IKGTVVLRKKNALNFNDAGSAFLDRMHELFGKHVSLQLI-SAVHADPGNGSKGKLGKPAILE-WTSSKTWISVEEAAVNITFD-WD-ESI : 121
CaLOX4    : -VRGTVVLMKKNVLDLTDVGASFLDRVHEVFGKGVSLQLI-SADHAEPGNGCKGKLGKPAFLENWVSTLTSISAGDATFNVTFD-WD-ESM : 128
CaLOX5    : -INGSVVLRKKTPLDLGSSCDVAHDEAYEILGHKVILQLISSSAHGDEG---KGKLGKPSHLA---VENKSGGNKDTCFNVTFE-WDHEGL :  92
CaLOX6    : -VKAVVTVQRTIRGT-NLSLSRGLDDIGDLFGRTILLCLV--AAELDPKTG-IEKPNIEAF-----ARRGRNVNGDKIYEAEFV-IP-EDF : 156
CaLOX7    : -VKVVVTVQKQV----NISLSRGLDDIGDLLGKSLLLWIV--AAELDPKTG-TEKPNIKAF-----AHRGKDVDGDTHYEADFSNIP-EDF : 148
CaLOX8    : DVKAVITLRKKMKEKISEKIEDQWVSLMNGIGRGILIQLI--SQDIDPVTK-SGKYA-ESYVRGW-FSKPSDHPYIVEYAANFT-VP-HDF : 165
CaLOX1(L) : -MKGTVVLMKKNALDFNDVNASFLDGVLEFLGKRVSLQLI-SSVHGDPANGLQGKRSKPAYLENWLTTRTPLVAGESAFDVTFD-WD-EDI : 106

CaLOX1    : GVPGAFTIKNSHINEFFLKSLILEDVPNHGKVHFVCNSWVYPSFRYKTDRIFFANQPYLPSETPEPLRKYRESELKTLRGDGTGKLEAWNR : 169
CaLOX2    : GTPGAITVTNKHQQEFFLESITIEGFA-CGPVHFPCNSWVQPKKDHPGKRIFFSNQPYLPNEMPAGLKSLREKELRDIRGDGTGVRKLSDR : 251
CaLOX3    : GVPGAFIIKNYHHSQFYLKTLTLQDVPGHGEVHFVCNSWVYPAHRYNYDRVFFSNKTYLPCNTPEPLQPYRNEELVNLRGTGSGMLKEWDR : 212
CaLOX4    : GFPGAFIIKNYHHSQLYLRTVVLEDVPGHGQLHFVCNSWVYPAHRYKYNRVFFANKTYLPSNTPEPLRPYREEELLSLRGSCSGMLKEWDR : 219
CaLOX5    : GVPGAFIIKNLNPTEFFLKSLSLT-LPSQDNLHFVCNSWVYPADKYDYDRIFFVNQALLPTETPEALRWYREAELLHLRGTGTGKLEEWDR : 182
CaLOX6    : GGVGAVLVENQHQKQMYVKNIVIDGFV-HGKVDITCNSWVHSKFDNPDKRIFFTNKSYLPSQTPSGVKRLREEELVTIRGDGFGERKIFER : 246
CaLOX7    : GEVGAILIENEHHKEMYVKNIVIDGFP-HGKVNITCNSWVHSKFDNPEKRVFFTNKSYLPSQTPSGVKRLREGELVTVRGDGVGVRKQFDR : 238
CaLOX8    : GCPGAIIITNLLDKEIHLVQIVVHGFN-EGPLFFSVNTWIHSQKDSPESRIIFQNQAYLPSQTPPGIKDLRREDLLSIRGNGKGERKLHER : 255
CaLOX1(L) : GVPGAFIINNLHFNEFFLKSLTLEDVPNHGKIHFVCNSWVYPAKRYKSERIFFANQAYLPHETPEPLREYREKELVTLRGDGNGKLEEWDR : 197
```

```
CaLOX1    : VYDYDVYNDLGNPDQGPEHVRTTLGGSADYPYPRRGRTSRPPTRTDPKSESRIPLLLSLDIYVPRDERFGHLKLSDFLTYALKSLVQFILP : 260
CaLOX2    : IYDYDIYNDLGNPDKGIDFARPKLGGNGNIAYPRRCRTGRVPMDTDMSAESRVEKP--NPTYVPRDEQFEESKMTTFSTSRLKAVLHNLIP : 340
CaLOX3    : VYDYAVYNDLG-------YDRPVLGGSKDHPYPRRGRTGRPLTKRDSSLESRLPLP-SLNIYVPRDECFNHVKFKDFLAYSATSIGRVIIP : 295
CaLOX4    : VYDYAFYNDLGFPDKGPEYVRPVLGGSKEYPYPRRGRTSRATKTDLNSESQLP-PLGLNIYVPRDERFTHVKLSDFLAYALKSLGQVLIP : 309
CaLOX5    : VYDYDVYNDFGDPDNSPLLARPVLGGSTEYPYPRRGRTGRPPSKADPKSESRLPQIASFAIYCPRDEKFSPLKLKDVLSNAQKAMAQLFSP : 273
CaLOX6    : IYDYDVYNDIGDPDGNDDGKRPVLGG-KKLPYPRRCRTG---------------------------------------------------- : 284
CaLOX7    : IYDYDVYNDLGDPDANDDCKRPVLGG-KELPYPRRCRTGRPRSKKDPLSESR-----SNSVYVPRDETFSEVKSLTFSGNTVHSVLHAVVP : 323
CaLOX8    : VYDYDVYNDLGNPDKSEDLARPLIGG-QERPYPRRCRTGRGPTKKDPLAERRIEKP--HPVYVPRDETFEEIKQNTFSAGRLKALLHNLVP : 343
CaLOX1(L) : VYDYAFYNDLGDPERGEAYARTILGGSAEFPYPRRGRTGRKSTKADPKSESRIPLLMSLDIYVPRDERFGHIKLSDFPTYALKSIVQFLIP : 288

CaLOX1    : ELHALFDGTPNEFDSFEDVLRLYEGGIKLPQGPLFKALTDAIPLEMIRELLRTDGEGILRFPTPLVIKDSKSAWRTDEEFAREMLAGVNPV : 351
CaLOX2    : SLMASISSNNHDFKGFSDIDSLYSEGLLLKLGLQDEVLNKLPLPKVVSSIKEG---DLLKYDTPKILSKDKFAWLRDDEFARQAIAGVNPV : 428
CaLOX3    : QTASLLARPFNEFISFKHVLGFYKDN----------AAEGCMPWKMFKV-------QFHKFPIPHVIKEDNSAWRTDEEFGREMLAGVNPL : 369
CaLOX4    : EIVALFDKTIDEFDSFEDVLKLYEGGIKLPDHHLNK-LRQCIPWEMLKELIRSDGEPPLKFPMPDVIKADRSAWRTDEEFGREMLAGVNPV : 399
CaLOX5    : QLAALGDVTLNEFNSFEDVLKAYEPG----------------------------APGYHKYPIPHVVRGDKSAWMSDEEFGREMLAGSNPV : 336
CaLOX6    : ---------------------------------------------------------------------DKFSWLRDEEFARQTLTGLNPY : 306
CaLOX7    : ALESVVSDPDLGFPHFPAIDSLFNVGVDIPGLGEKKGGLLNVIPRLFKAISDT-GKDVLLFETPQLLERDKFSWFRDVEFARQTLAGLNPY : 413
CaLOX8    : LIAATLSSSDIPFTNFTDIDKLYNDGFVLNDDKDLK--KNKFLSDTLDKVFSV-SKRLLKYEIPAIIKRDRFAWLRDNEFSRQALAGVNPV : 431
CaLOX1(L) : EFQALFDSTPGEFDSFEDVLRLYEGGIKLPQGPFLKALTDSIPLSILKEIIRTDGEGKFKFPTPQVIQADKSSWRTDEEFAREMLAGVNPV : 379

CaLOX1    : IISRLQEFPPKSKLDPNVYGNQDSTITAEHIQDKLDG-LTIDQAINNNKLFILNHHDILTPYLRRINTTT-TKTYASRTLLFLQDNGSLKP : 440
CaLOX2    : TIERLQVFPPVSKLDPEIYGPQESALKEEHIRGHLNG-MTVQEALDANKLFIVDYHDVYLPFLDRINALDGRKAYATRTIFFLSSLGTLKP : 518
CaLOX3    : IIQRMQEFPPTSKLNPEVYGNQTSKITREHIETYMDG-LTVDDAIKHNRLFILDYHDILMPYLKRVNSTT-TKIYASRTLLLLRDDGTLQP : 458
CaLOX4    : IIRRLQEFPPASKLDPKVYGNQTSSITREHIEKNLDG-LTVDEAIEYNKLFILDHHDALMPYLRRINTTK-TKTYASRTLLFLQDNGTLKP : 488
CaLOX5    : CIRGLKEFPPTSKLDPKIYGDQTSKITREQIQSQLGG-LTIEKAMETNRMFILNYHDIVMPYARKLNETP-SKIYATRTVLFLQNDGTLKP : 425
CaLOX6    : SIRLVTEWPLKSKLDPKAYGPPESAIAKEMIELEIEGLMTVEEAIEQKKLFILDYHDLLLPFVNKVNELKGTVLYGSRTLFFLTPNGTLRP : 397
CaLOX7    : SIRLVTEWPLKSKLDPEVYGPPESAITKELIELEIAGFMTVEEAVAQKKLFILDYHDLLMPYVNKVNELKGTVLYGSRTLFFLTPDGTLRP : 504
CaLOX8    : NIELLREFPIVSKLDPAVYGPPDSAVTRDLIEQELNG-LSVEKAIEEKRLFILDYHDMLLPFIGKMNSLLGRKAYASRTLFFYTSRGVLKP : 521
CaLOX1(L) : IISRLQEFPPKSKLDTEVYGNQNSTITKEHIENALDG-LTIDDAIKTNRLYILNHHDMLMPYVRRINTTN-TKLYASRTLLFLQDDGTMKP : 468
```

```
CaLOX1    : LAIELSLPHPDGDQFGVISKVYTP-SDQGVESSIWQLAKAYAAVNDSGVHQLISHWLNTHAVIEPFVIATNRQLSVLHPIHKLLYPHFRDT : 530
CaLOX2    : IAIELSLPQTGPSS--RSKRVVTP-PVDATGNWMWQLAKAHVCSNDAGVHQLVNHWLRTHACLEPFILAAHRQLSAMHPIYKLLDPHMRYT : 606
CaLOX3    : LAIELSLPHPQGDKHGSSSQIFTPCSDESAEGYVWHLAKAYVAVNDSGYHQLVSHWLNTHAVIEPFIIATNRQLSVLHPIYKLLQPHFRDT : 549
CaLOX4    : LAIELSLPHPQGDKHGATSLVFTP-ADEGVEGTVWQLAKAYAAVNDSGYHQLISHWLNTHAVIEPFVIATNRQLSVLHPIFKLLQPHFRDT : 578
CaLOX5    : LAIELSLPHPDGDQFGAISKVLTP-AVTGVEYGLWQIAKAFVSVNESGIHQLISHWLHTHGSVEPFVLATHRQLSVLHPIYKLLHPHFRDT : 515
CaLOX6    : LAIELTRPPVDDKP--QWKEVYCPSTWHATGAWLWRTAKAHVLAHDSGYHQLVSHWLRTHCATEPYIIASNRQLSAMHPIYRLLFPHFRYT : 486
CaLOX7    : LAIELIRPPVDGKP--QWKRVYCP-TWHATGAWLWKLAKAHVLAQDSGYHQLISHWLRTHCCTEPYIIATNRQLSAMHPIYRLLHPHFRYT : 592
CaLOX8    : IIIELSLPPTPSSP--RNKRIFSH-GHDATNHWIWNLAKAHVCSNDAGIHQLVNHWLRTHACMEPYIIATHRHLSSMHPIYKLLHPHMRYT : 609
CaLOX1(L) : IAIELSLPHPDGDELGAVSKVYTP-ADRDVEGTIWQLAKAYVAVNDSGVHQLISHWLNTHAAIEPFVIATNRQLSVLHPIHKLLHPHFRDT : 558

CaLOX1    : MNINALARQILINAGGVLESTVFPSKYAMEMSAVVYKD-WVFPDQALPADLIKRGIAVEDSSSPHGVRLLIQDYPYAVDGLEIWSAIKSWV : 620
CaLOX2    : LEINGLARQSLINADGVIEACFTPGRYCMEISAAAYKN-WRFDLEGLPADLIRRGMAVPDSTQPHGLKLLIEDYPYAADGLMIWAAIEGWI : 696
CaLOX3    : MNINALARHILISAGGVLELTVFPSKYALEMCSSIYKN-WVFTEQALPADLIKRGVAVPDPSEPHGLKLLIKDYPYAVDGLEIWSAIEGWV : 639
CaLOX4    : MYINALARQILINAGGILERTVFPAKYAMEMSSIVYKN-WVFTEQ--------RGVAVPDSSQPYGLKLLIEDYPYAVDGLEIWEAIEAWV : 660
CaLOX5    : MHINALARQAILHGGGIVERTVFPGAHCMELTSIAYKD-WVFPDQALPAXXXLNLLRVEDPASEHGVRLLIEDYPYAVDGLEIWSAIKSWV : 605
CaLOX6    : MEINATAREALINANGAIETSFSLGKYSMELSAVAYDLEWRFDRQALPEDLLSRGLAEEDPNAPYGLKLTIEDYPFANDGLVLWDILKQWV : 577
CaLOX7    : MEINALAREALINANGIIESSFFPGKYALELSAVAYGLEWRFDQEALPANLISRGLAVEDPNEPHGLKLTIEDYPFANDGLVLWDTLKQWV : 683
CaLOX8    : LEINALARQSLINGGGVIEACFSPGKYSMEISSAAYKSMWQFDMEALPADLIRRGMAVEDPSMPLGVKLVIEDYPYAADGLLIWSAIKEYV : 700
CaLOX1(L) : MNINALARQILINAGGVLELTVFPSKYAMEMSAVVYRN-WVFPEQALPVDLVKRGVAVEDSSSPHGVRLLIQDYPYAVDGLEIWSAIKIWV : 648

CaLOX1    : TEYCNVYYKSNEDILKDNELQEWWKELREVGHGDKKDAPWWPEMESPEDLIESCTIIIWIASALHAAVNFGQYPYAGYLPNRPTVSRRFMP : 711
CaLOX2    : RDYVNHYYQDSAQVCNDRELQAWYTESINVGHADLRNEDWWPTLATPEDLISILTTLIWLASAQHAALNFGQYPYGGYVPNRPPLMRRLIP : 787
CaLOX3    : NDYCSLYYTTDDMIRDDTELQSWWTEVHDEGHGDLKDEKWWPQMQTKAELIQICTTIIWVASALHAAVNFGQYPYAGYLPNRPTISRRFMP : 730
CaLOX4    : DDYCSFYYSTDDMIRGDSELQSWWKEVRDEGHGDLKDEPWWPQMQTRAELVQACTIIIWTASALHAAVNFGQYPYAGYLPNRPTVSRRFMP : 751
CaLOX5    : RDYTSLYYKTDDVILQDSELQAWWKEIREVGHGDKSGEPWWPKMQTREELVHSLTIIIWMASALHAAINFGQYPYGGFAPNRPGMCRRLIP : 696
CaLOX6    : TAYVNHYYPQTNLIESDIELQDWWSEIKNVGHGDKKDEPWWPELKTPNDLIGIVTTIIWVASAHHAAVNFGQYSYAGYFPNRPTIARTKMP : 668
CaLOX7    : TAYVNHYYPQTNLVESDIELQAWWSEIKNVGHADKKDEPWWPELKTPNDLIGIITTIVWVTSGHHAAVNFGQYSYAGYFPNRPTIARSKMP : 774
CaLOX8    : ESYVEHYYSEPNSVTSDVELQGWWNEIKNKGHPDKKNEPWWPKLVTKEDLSGILTTMIWIASGQHAAINFGQYPFGGYVPNRPTLMRKLIP : 791
CaLOX1(L) : TEYCNFYYKSDESVLKDDELQAWWKEVREEGHGDKKDEPWWPKMQTRQELIDSCTIIIWIASALHAAVNFGQYPYAGYLPNRPTLSRRFMP : 739
```

```
CaLOX1    : --EPGTPEYEELKTNPDKAFLKTITAQFQTLLGVSLIEILSRHTSDEIYLGQRESPE-WT-KDKEPLAAFDRFGKKLTEIENHIIQRNGDQ : 798
CaLOX2    : --DENDPEYAVFLADPQKYFFSALPSLLQATKFMAVVDTLSTHSPDEEYLGERHQPSTWT-GDAEIVEAFYEFSAEMRRIEKEIDEKNVNT : 875
CaLOX3    : --EPGTPEYAELESNHELAYLKTITAQFQTLLGISLIEMLSMHSTDEIYLGQRDTPE-WT-SDTQPRHALERFRDKLIEIEKSIMDRNNDS : 817
CaLOX4    : --EPGTAEYAELESNPDLAYLKTITAQFQTLLGVSLIEILSRHSSDEIYLGQRDNPE-WT-SDIQPRQSFQRFHDRLVDVEKKIVERNNDS : 838
CaLOX5    : --DPGTADYEELKTNPVKGYLKTISPQFQTLIGIAVLEVLSIHSSDEYFLGQREAAAEWT-EDMEALKAFERFGKKLAQIEEKITMMNNDE : 784
CaLOX6    : TEDPTDEEWENFLNKPEEALLKCFPKQIQATIVMAVLDVLSNHSPDEEYVGKNIEPY-WS-EDPIINTAFEVFSGKLKELEGIIDARNADC : 757
CaLOX7    : TEDPTDEEWECFLNKPEEALLKCFPSQIQATKVMAILDVLSNHSPDEEYLGETIEPY-WA-EDPVIKAAFEVFSGKLKELEGIIDARNADP : 863
CaLOX8    : --HEDDPSYENFILHPEYTFLASLPTQLQATKVMAVQDTLSTHSADEEYMCQLHEIQRFSIYDHEVLKVFERFSAKLKEIENTINQRNKDI : 880
CaLOX1(L) : --EPGTPEYEELKTNPDLAYLKTITPQLQTLLGISLIEILSRHTSDEVYLGQRDSSE-WT-KDQEPLAAFERFGKKLSEIEDQIVQMNGDE : 826

CaLOX1    : ILKNRSGPVNAPYTLLFP-----TSEGGL-TGKGIPNSVSI : 833
CaLOX2    : KLRNRCGAGVLPYELLAP-----SSGPGV-TCRGVPNSVSI : 910
CaLOX3    : TFKNRNGPVQMPYTLLCPNASGDNSATGL-TGKGIPNSVSI : 857
CaLOX4    : RWKNRNGPVKVPYMLLYPNASGDNSESGL-TVKGIPNSVSI : 878
CaLOX5    : KLKNRTGPVKMPYTLLYP-----TSEPGLVTAKGIPNSISI : 820
CaLOX6    : NLRNRNGAGIVPYELLKP-----FSGPGV-TGKGVPYSISI : 792
CaLOX7    : KLMNRNGAGVVPYELLKP-----FSGPGV------------ : 887
CaLOX8    : RLKNRSGAGVPPYELLLP-----TSGPGV-TCRGIPNSISI : 915
CaLOX1(L) : NWKNRSGPVKVPYTLLFP-----TSEEGL-TGKGIPNSVSI : 861
```

Figure S1: Multiple sequence alignment of protein sequences of pepper LOXs. The alignment was performed with MUSCLE and viewed in GeneDoc. Black and gray background possessing amino acids represents identical or similar amino acids. The green arrows depicts the five amino acids essential for Fe (iron) binding. Red frame depicts Val or Phe residue, indicative for LOX protein to possess 9- or 13-LOX activity, respectively. The Arg and Ala residue determinant for inverse substrate orientation and stereospecificity of LOXs are shown in purple and orange hexagon, respectively.
